# Effects of sediment smothering on the sponge holobiont with implications for dredging management

**DOI:** 10.1038/s41598-017-05243-x

**Published:** 2017-07-14

**Authors:** Mari-Carmen Pineda, Brian Strehlow, Miriam Sternel, Alan Duckworth, Joost den Haan, Ross Jones, Nicole S. Webster

**Affiliations:** 1 Australian Institute of Marine Science (AIMS), Townsville, QLD and Perth, WA Australia; 2 Western Australian Marine Science Institution, Perth, WA Australia; 3 Centre for Microscopy Characterisation and Analysis, School of Plant Biology, and Oceans Institute, University of Western Australia, Crawley, WA Australia; 4 University of Bremen, Bremen, Germany; 5 Max Plank Institute for Marine Microbiology, Bremen, Germany

## Abstract

One of the ways dredging can affect benthic habitats is through high levels of sediment deposition, which has the potential to smother sessile organisms such as sponges. In order to provide pressure-response values to sedimentation and tease apart the different cause-effect pathways of high turbidity, 5 sponge species, including heterotrophic and phototrophic nutritional modes, were exposed for up to 30 d to multiple sediment deposition events, each of which resulted in an initial covering of 80–100% of the surface of the sponges in a layer ~0.5 mm thick. The response of the sponges was examined using a suite of different response variables including growth, respiration, lipid content, community composition of the microbial symbionts, and maximum quantum yield and chlorophyll content of the phototrophic symbionts. Different species showed different mechanisms of sediment rejection and different patterns of sediment clearance. All species survived the treatments, were able to tolerate high levels of partial covering of their surfaces, and for most species the treatment did not alter the health of the sponge holobiont. Results from this study will guide interpretation of experiments examining the combined effects of all three dredging-related pressures, and aid the development of water quality thresholds for impact prediction purposes.

## Introduction

The growing natural resource industry and global expansion of maritime transport have increased the need for capital and maintenance dredging over the past decades, with millions of cubic metres of sediments being displaced annually around the world^1–4^. The release of sediments into the water column by dredging and dredging activities such as spoil disposal, temporarily increases water turbidity (cloudiness)^5, 6^. The increased suspended sediment concentrations (SSCs) can clog the feeding apparatus of sessile filter feeders^7^ as well as reduce the quantity and quality of benthic light for phototrophs^3, 5–9^. Sediments can remain in suspension for many hours, depending on their particle size and the local hydrodynamic conditions, but will ultimately fall back out of suspension. High sediment deposition rates occur during dredging programs as high SSCs are created in sea-states where ambient hydrodynamics cannot support the load. When sediments deposition rates become too high, sediments can begin to smother benthic organisms and there are many *in situ* observations of this occurring in dredging projects (see photographs in refs 6, 10 and 11). In order to improve the ability to predict and manage the impacts of dredging it is necessary to understand the tolerance of ecologically important habitat-forming species to all potential hazards associated with dredging. In this study, we examine the response of five sponge species (including heterotrophic and phototrophic nutritional modes), to repeated smothering by sediments, complementing earlier studies of the effects of suspended sediments^7^ and light attenuation^8^ on the same species.

Sponges (Porifera) are sessile filter-feeding organisms that play important roles in marine ecosystems, including substrate consolidation, habitat provision, seawater filtration, and linking the benthic and pelagic environments via energy transfer^12, 13^. Sponges are abundant and highly diverse, being the dominant fauna in many regions including some coral reefs, inter-reefal habitats and deep water environments (e.g. refs 14–18). However, sponge assemblages can also be sensitive to global and local pressures including dredging-associated increases in sediment suspension and deposition^7, 19–22^. Sponges harbour dense and diverse symbiotic microorganisms which are known to contribute to the health, fitness and nutrition of their hosts^23^. In some species, these symbiotic microbes can comprise 40% of the sponge volume and due to their functional importance, we do not consider the symbionts independently, but rather consider the sponge as a stable host-microbe consortium termed the ‘sponge holobiont’^23^. Sponge symbionts tend to be highly host specific and are generally stable across broad geographic and environmental gradients^24^, although loss of symbiont function and holobiont destabilisation has been reported following acute environmental stress^25^. Importantly, most sponge species are unable to survive environmentally induced symbiont disruption^8, 26, 27^.

Whilst most sponge species obtain food by filtering particles from the surrounding seawater, >100 different sponge species also supplement their heterotrophic feeding with the autotrophic metabolism of dinoflagellates or microbial photosymbionts^28–32^. Depending on their degree of nutritional dependence on symbiont primary production, sponges are described as either ‘phototrophic, ‘mixotrophic’ or ‘heterotrophic’. Notably, some species exhibit considerable flexibility in their feeding strategy and are able to alter their nutritional mode depending on prevailing environmental conditions^7, 8, 33–35^.

Sediment deposition may differentially affect sponges depending on their morphology, their dependence on heterotrophic versus phototrophic feeding, the stability of their microbiomes as well as other physiological traits^20^. Sponges known to tolerate sediment stress include endopsammic species (living partially buried within sediments), fast growing species with morphological plasticity and species with growth forms that have exhalant openings on apical body parts^20, 36, 37^. Sponge morphology is a particularly important factor in sediment tolerance, with erect and upright species generally less prone to sediment smothering than encrusting, massive, cups and plate-like morphologies^19, 20^. Sediment smothering may also block light from reaching the sponge surface, thereby adversely affecting the photosynthetic ability of microbial symbionts. Sediments could also cause temporary closure of the incurrent openings (ostia), affecting water pumping and nutrient uptake by the sponge^7, 20^. These effects can compromise both phototrophic and heterotrophic feeding, reducing flow of oxygenated seawater to the sponge mesohyl and having flow on consequences for host energetics, health and reproductive output^7, 19–22, 38^. In addition, while some species may experience a sediment induced shift in the microbiome which enables them to acclimate to the new environmental conditions, other species may undergo microbial changes that destabilise the holobiont and trigger high levels of bleaching and mortality^7, 8^.

Sponges have a number of cleaning mechanisms to remove sediments from their surfaces and reduce the risk of clogging of the internal canal system. These include mucus production, tissue sloughing, self-cleaning surfaces, sediment removal by epibionts, selective rejection of inhaled particles and the use of water jets to unblock inhalant areas^20, 39–41^. Some of these self-cleaning strategies are energy-depleting processes which may not be sustainable in the longer term^19, 20^. Once the capacity to remove sediments becomes overwhelmed, sediments will remain on the sponges’ surface and accumulate. While hard corals are known to be very susceptible to accumulation and smothering, with effects occurring within a few days^42^, the sensitivity of sponges to sediment smothering is not well known. Close to dredging operations, sponges will be exposed to high sediment deposition levels, but this will invariably occur in combination with elevated SSCs and the associated high levels of light attenuation^6^. Simultaneous testing of all dredging-related pressures in combination prevents isolation of the specific effects of sediment deposition alone, precluding identification of cause-effect pathways (mechanisms) and hindering the establishment of potential bio-indicators. The effects of elevated SSCs and low light on 5 sponge species (spanning heterotrophic and phototrophic nutritional modes) has recently been described using a suite of physiological and molecular assays^7, 8^. In this study, we use the same assays to examine the response of the same species to repeated sediment deposition (i.e. smothering events) over an extended (up to 30 d) period.

## Results

### Physical parameters

**Figure 1:**
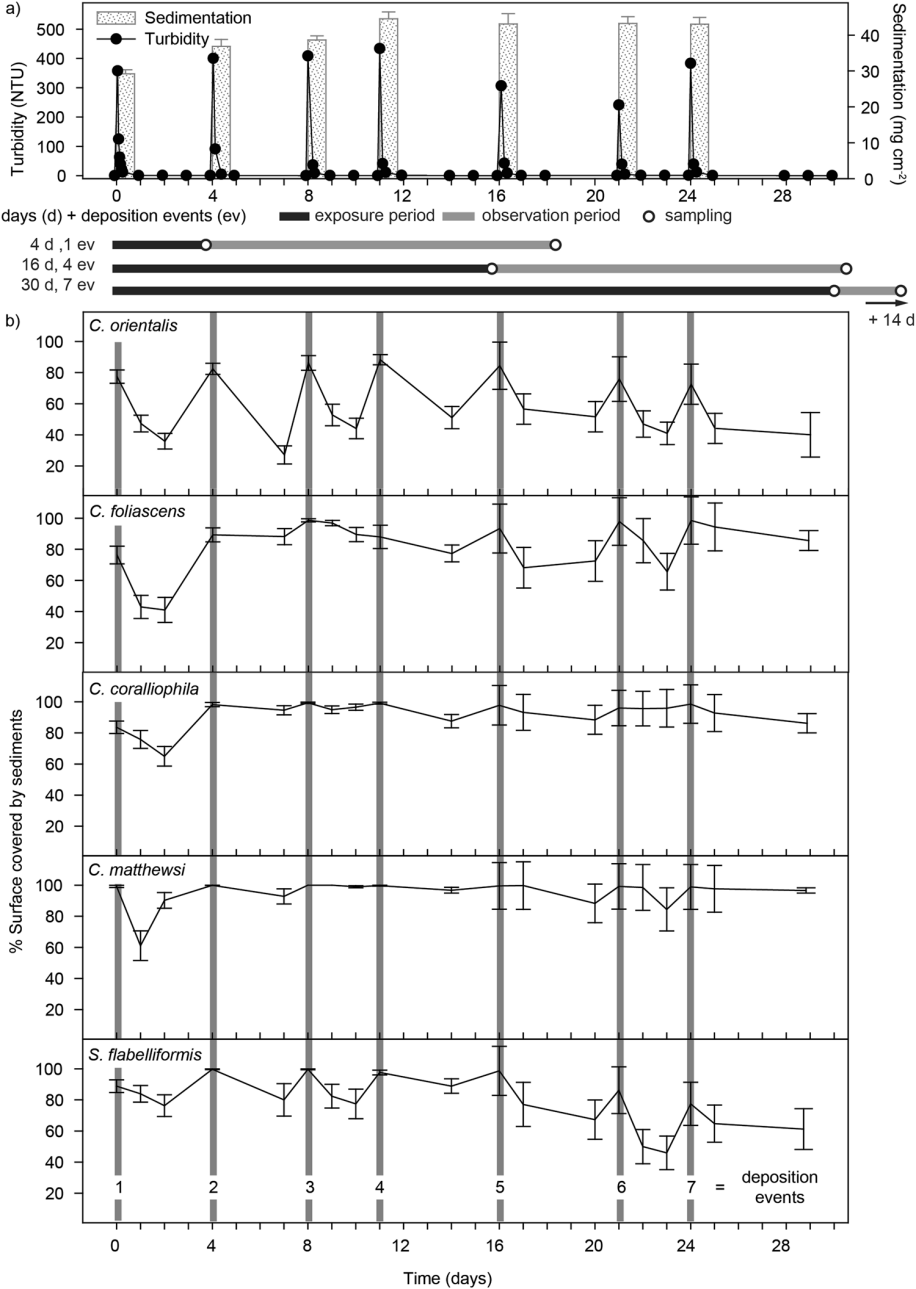
Sediment behaviour across the experiment. Mean values (±SE) of (**a**) sedimentation and turbidity levels in the smothering treatments. Deposition events occurred every ~4 d with sponges being exposed to either 1, 4 or 7 deposition events and then transferred to clean seawater (for a 14 d observation period), and (**b**) percentage of sponge surface covered by sediments for each species, throughout the experiment.

In order to keep a continuous layer of sediment over sponges within the smothered treatment, sediment deposition events ranging from 29 ± 2 to 44.5 ± 1.9 mg cm^−2^, were created on days 0, 4, 8, 12, 16, 21 and 24 (mean ± SE, Fig. 1a). Sedimentation in control tanks ranged between 0.1–0.2 mg cm^−2^. No significant differences occurred between replicate tanks within either the control or smothered treatment at each monitoring time separately (ANOVA: *P* > 0.1). However, significantly higher sedimentation levels were obtained after the initial pulse (Day 0, Fig. 1a, ANOVA: *P* < 0.001), due to the resuspension of previously settled sediments once pumps were turned on to ensure homogenous turbidity after each pulse.

Turbidity values in the tanks during the injection of the sediment (to create the sedimentation events) reached ~430 NTUs, although turbidity rapidly decreased to <10 NTUs within 6 h, and for ~95% of the time turbidity levels were <1 NTU in both the smothered and control treatments (Fig. 1a). Hence, despite the high turbidity levels observed immediately after the sediment pulse, sedimentation/smothering remained the primary dredging-related pressure being applied to sponges in this study. No significant differences in turbidity levels were found between replicate tanks within a treatment (ANOVA: *P* > 0.999).

### Sediment clearance rates and removal mechanisms

The sedimentation events typically resulted in an initial coverage of 80–100% of the sponge surfaces across all species in a layer of sediment ~0.5 mm thick. The percentage of sponge surface covered by sediments often decreased on the days following the events (Fig. 1b). The pattern of sediment clearance differed among species, with an overall significant decrease in surface sediments after the initial event, but the ability to clear the sediment decreased after subsequent events in *Carteriospongia foliascens*, *Cymbastela coralliophila* and *Coscinoderma matthewsi* (*P* < 0.0001, <0.001 and <0.001, respectively) (Fig. 1b). In contrast, *Cliona orientalis* and *Stylissa flabelliformis* maintained similar clearance rates throughout the experiment. *C. orientalis* was more effective at removing sediment than *S. flabelliformis*, with sediment cover significantly decreasing from 80 to 20–40% at 3 d post-deposition, while the latter species showed higher variability and >50% of its surface remained covered throughout the experiment (*P* < 0.0001 and <0.0001, respectively) (Fig. 1b). Sponges demonstrated an array of different mechanisms for coping with sedimentation, such as sediment sloughing via spicule hispidity and removal of surface sediment by infauna such as brittle stars (Supplementary Fig. S1). Mucus production was also observed in *S. flabelliformis* and *C. foliascens* and the peeling of the sediment-impregnated mucus resulted in a comparatively sediment-free surface in these species (see Supplementary Fig. S1).

### Sponge health, growth and necrosis

All sponges survived the 4, 16 and 30 d treatments and subsequent observational phase, and necrosis was not observed in any species. The partial sediment covering caused no visible signs of stress in most sponges, with the exception of some (~7%) partial bleaching in the phototrophic species *C. orientalis* in the 16 d and 30 d treatments, although this species recovered its initial appearance by the end of the observational phase. No visible effects of sediment covering were observed in any of the other species at the end of the experiment.

**Figure 2:**
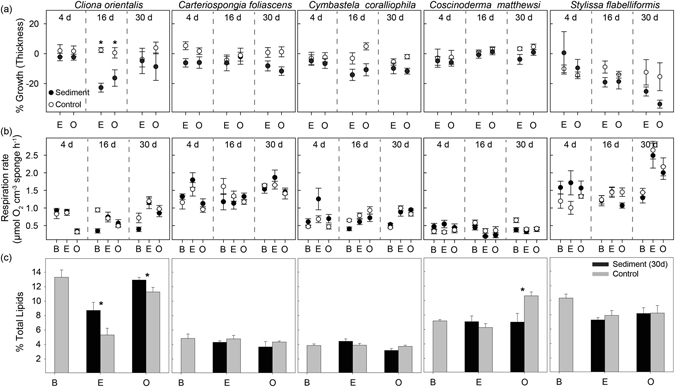
Physiological responses of sponges to sediment smothering. (**a**) Percentage of growth (based on sponge thickness), (**b**) Mean respiration rates (µmol O_2_ cm^−3^ sponge h^−1^) for all species and treatments (control vs. smothered for 4, 16 or 30 d), (**c**) Percentage of sponge biomass comprised of lipids, for all species exposed for 30 d, where ‘B’ is before sediment addition, ‘E’ is after the exposure period and ‘O’ is at the end of the observational phase. All data are mean ± SE and asterisks show statistically significant differences between smothered and control sponges (t-tests: P < 0.05).

Analysis of growth rates based on tissue thickness measurements showed negative growth in most sponges covered by sediments for 16 and 30 d, although the response was species-specific and significant differences between controls and smothered sponges were only observed in the phototrophic species *C. orientalis* (Fig. 2a).

### Respiration Rates

Overall sponge respiration rates were not significantly different between treatments (control vs. smothered) before the deposition events (t = 0), with species-specific responses following sediment exposure (Fig. 2b). In general, a rapid increase in respiration rates was observed in the sponges covered and partially covered for 4 d, although this was not significantly different to the controls (Fig. 2b). Respiration rates in *C. foliascens* and *C. coralliophila* smothered for 4 d were significantly higher than before exposure (Fig. 2b, Table 1B). A similar response was observed in *C. orientalis* and *C. coralliophila* after 16 d and in *S. flabelliformis* after 30 d (Table 1B). However, similar respiration rates between controls and smothered samples were observed under the longer term treatments (16 and 30 d) (Fig. 2b).

**Table 1:** ANOVA tables and summaries of linear mixed models testing the effects of smothering on the physiological responses of sponges.

Source	*df*	*C. orientalis*		*C. foliascens*		*C. coralliophila*		*C. matthewsi*		*S. flabelliformis*	
		*F*	*P*	*F*	*P*	*F*	*P*	*F*	*P*	*F*	*P*
**(A) Growth**											
Treatment	5	5.46	0.008	1.735	0.201	2.556	0.085	1.666	0.2171	1.797	0.188
Time	1	0.215	0.645	0.001	0.974	1.398	0.243	0.945	0.336	2.600	0.114
Treat. × Time	5	0.442	0.817	0.588	0.709	0.964	0.449	0.226	0.950	0.244	0.941
Error	48										
**Significant Pairwise Multiple Comparisons (Tukey)**											
Treatment		16 d-C > 16 d-S 4 d-S > 16 d-S									
**(B) Respiration Rates**											
Treatment	5	2.154	0.12	2.558	0.084	2.465	0.093	2.056	0.144	6.530	<0.01
Ind.(Treatment)	30										
Time	2	32.269	<0.001	8.345	<0.001	64.397	<0.001	8.885	<0.001	8.208	<0.001
Treat. × Time	60	11.453	<0.001	2.097	0.039	6.447	<0.001	2.168	0.033	2.823	<0.01
Error	107										
**Significant Pairwise Multiple Comparisons (Tukey)**											
Time				B > O		B < O		B > E, O			
Time within 4 d-S		B, E > O		B < E > O		B, O < E					
Time within 16 d-S		B < E				B < E, O		B > E, O			
Time within 30 d-S		B < E > O				B < E, O				B < E, O	
Treatments within E				16 d-S < 30 d-S		4 d-S > 16 d-S		4 d-S > 16 d-S			
Treatments within O										16 d-S < 30 d-S	
**(C) Percentage of sponge biomass comprised of lipids**											
Time	1	39.04 3	<0.001	1.448	0.295	7.882	0.047	8.442 8	0.010	0.692	0.418
Treatment	1	9.888	0.034	1.444	0.247	0.004	0.951	1.887	0.242	0.144	0.724
Treat. × Time	20	1.187	0.292	0.039	0.846	4.642	0.013	8.836	0.009	0.154	0.700
Error	23										
**Significant Pairwise Multiple Comparisons (Tukey)**											
Time		E < O						E < O			
Treatment		C < S									
Treat. × Time						[S] E > O		[C] E < O; [O] C > S			

(A) Percent growth rate based on thickness measures, (B) Respiration rates and (C) Percentage of sponge biomass comprised of lipids. Tukey tests have been performed for significant pairwise multiple comparisons. Abbreviations: ‘B’ for before sediment pulse (T = 0), ‘E’ for end of experimental phase, ‘O’ for end of observational period, ‘C’ for controls, ‘S’ for smothered, and ‘4 d’, ‘16 d’ and ‘30 d’ for time smothered.

### Lipids

Total lipid content (representing 2–16% of total sponge biomass) was highly stable in all studied species across the 30 d exposure period and subsequent 14 d observational period (Fig. 2c). Overall, smothered sponges did not have lower lipid concentrations. In contrast, *C. orientalis* had a higher percentage of lipids per biomass in the sediment treatment (Fig. 2c, Table 1C). In addition, significantly lower lipid content was detected in sediment-exposed samples of *C. coralliophila* after the observational phase, in comparison with samples at the end of the exposure time (Table 1C). *C. matthewsi* had significantly higher lipid content in control samples during the observational period. *C. foliascens* and *S. flabelliformis* did not show any significant change in lipid content throughout the experiment (Fig. 2c, Table 1C).

### Hyperspectral imaging

**Figure 3:**
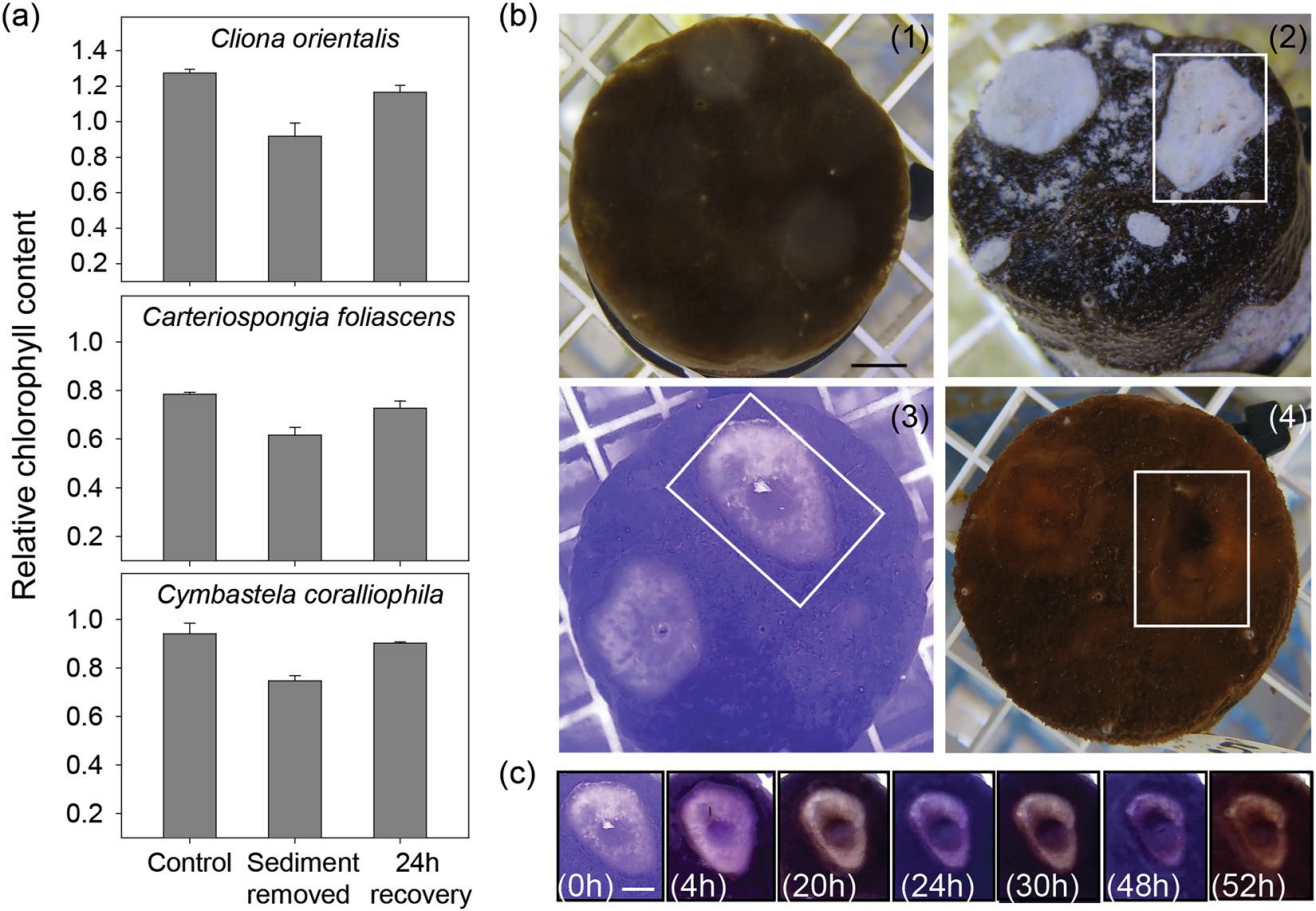
*In-situ* chlorophyll assessment using hyperspectral and time-lapse imaging. (**a**) Relative chlorophyll content of the three phototrophic sponge species smothered with sediments for 8 d (mean ± SE); (**b**) Bleaching recovery in *C. orientalis* following treatment with sediments for 30 d: (1) Before exposure to sediments (T = 0), (2) During smothering, (3) Bleached area (note white rectangle) evident following sediment removal, (4) Colouration after 6 d recovery; (**c**) Time-lapse recovery following sediment clearance (0 h) until 52 h later (scale = 1 cm).

Hyperspectral imaging revealed that the relative chlorophyll content derived from reflectance spectra over the surface of the specimens differed significantly between species, with higher chlorophyll in *C. orientalis*, followed by *C. coralliophila* and *C. foliascens* (ANOVA: *P* < 0.001). Hyperspectral scans of smothered sponges yielded negligible relative chlorophyll contents (0.34 ± 0.1, 0.08 ± 0.08 and 0.04 ± 0.01 in *C. orientalis*, *C. foliascens* and *C. coralliophila*, respectively, mean ± SE). However, the chlorophyll signal became clear once sediment was removed and significantly lower chlorophyll content was detected in smothered sponges than in control individuals for all species (ANOVA: *P* < 0.05, Fig. 3a, Supplementary Table S1). However, after 24 h recovery from sediment exposure, the relative chlorophyll content was not significantly different from the control group for any species, indicating rapid recovery potential (Fig. 3a).

**Figure 4:**
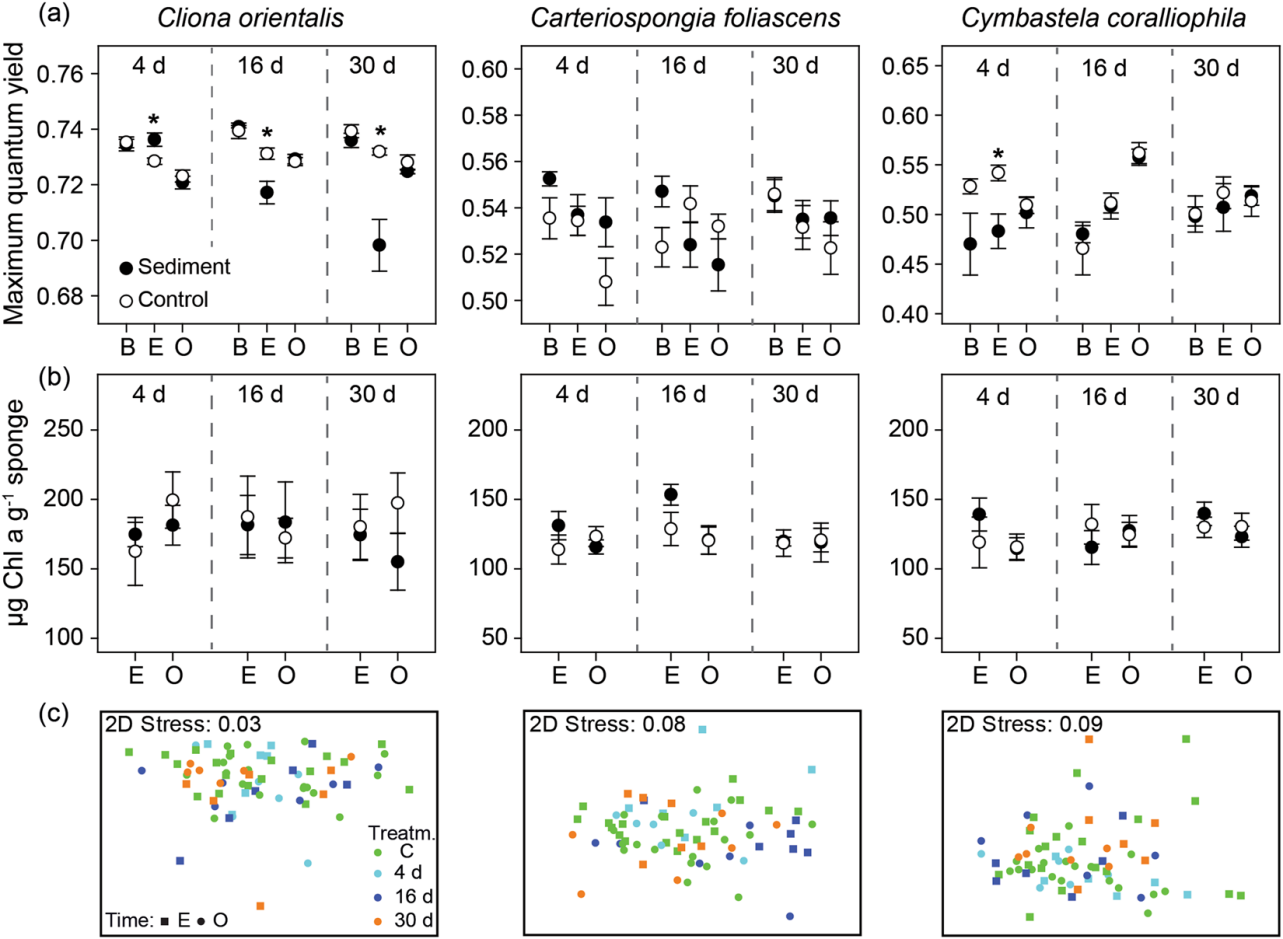
Responses of photosymbionts to smothering treatments. (**a**) Mean values (±SE) of maximum quantum yield, (**b**) Mean values (±SE) of Chl a, and (**c**) Non-metric Multi-Dimensional Scaling (nMDS) of all photopigments retrieved by spectrophotometry, for the 3 phototrophic species and for all smothering treatments (4, 16 and 30 d smothered vs. controls), before exposure (**b**) and through the experimental (**E**) and observational periods (**O**). Asterisks show statistically significant differences between controls and smothered samples (Table 2) (t-tests: *P* < 0.05).

The recovery response was observed in greater detail for *C. orientalis* (Fig. 3b,c). This species appeared to be capable of manipulating and consolidating the settled sediments into discrete patches which were typically observed in the central areas and usually in surface troughs or depressions (Fig. 3b-2, see also Supplementary Fig. S1). Following sediment removal, the areas under the sediment appeared bleached (Fig. 3b-3). Time-lapse photography revealed near full recovery of the bleached patches within 52 h (Fig. 3c), and full recovery after 6 d (Fig. 3b-4). The bleached area recovered from the centre outwards as well as from the borders inwards, indicating that *Symbiodinium* sp. cells are migrating not only from the unbleached tissue but also possibly from a population residing deeper within the sponge where the substrate has been excavated.

### Chlorophyll fluorescence

The photosynthetic efficiency of sponge symbionts was only affected by sediment in *C. orientalis*, with significantly lower quantum yields from smothered samples at the end of the 16 and 30 d experimental period (Fig. 4a, Table 2A). This result is consistent with the observed bleaching in this species. Maximum quantum yield values returned to control levels after the 14 d observational period and colouration also recovered during this period (Fig. 4a). Increased yield values after only 4 d of smothering indicates an initial photoacclimation response in this species, although longer term sediment exposure appears to damage the photosystems (Fig. 4a). There were few significant changes in yield values among the other two phototrophic species, *C. foliascens* and *C. coralliophila*, with only minor fluctuations through the experiment and observational periods (Fig. 4a, Table 2A).

**Table 2:** ANOVA tables and summaries of linear mixed models of the effects of smothering on the photosymbionts.

Source	*df*	*Cliona orientalis*		*Carteriospongia foliascens*		*Cymbastela coralliophila*	
		*F*	*P*	*F*	*P*	*F*	*P*
**(A) Maximum quantum yield**							
Treatment	5	5.10	0.01	0.850	0.542	1.777	0.192
Time	2	46.00	<0.001	8.860	<0.001	11.832	<0.001
Treatm. × Time	10	10.10	<0.001	1.780	0.084	2.430	0.017
Error	60						
**Tukey**							
Time		O < B				B < O; E < O	
Time within 4 d-S		B, E > O				B < O	
Time within 16 d-S		B > E, O; E < O		B > O		B < O	
Time within 30 d-S		B > O					
Treatment within E		4 d-S > 16 d-S, 30 d-S 30 d-C > 30 d-S					
**(B) Chl a**							
Treatment	5	0.250	0.932	0.847	0.543	0.450	0.778
Time	1	0.149	0.701	2.114	0.153	1.074	0.305
Treatm. × Time	5	0.496	0.777	1.373	0.2511	0.684	0.638
Error	60						
**(C) PERMANOVA of all pigment data**							
Treatment	5	0.53843	0.8182	2.2158	0.0332	0.4343	0.1679
Time (Treatment)	6	0.6232	0.7694	1.199	0.299	1.6895	0.078
Error	60						
Pair-wise Tests				4 d-C, 16 d-C, 4 d-S ≠ 16 d-S ≠ 30 d-S			

(A) The effects of treatment and time on maximum quantum yield throughout the experiment with Tukey tests performed for significant pairwise multiple comparisons, (B) The effects of treatment on Chl a concentrations across time (at the end of the experimental (E) and observational (O) periods) and treatment, with Tukey tests performed for significant pairwise multiple comparisons, and (C) Two-way PERMANOVA of all pigment data (Chl a, b, c, d, total Chl and carotenoids) with sediment treatment and time as factors, for the 3 phototrophic species (sediment treatments: 4, 16 and 30 d smothered vs. controls).

### Pigment analysis

Total chlorophyll was found to be highly correlated to Chl a in all 3 phototrophic species (R^2^ = 0.999, 0.998, 0.952 and *P* < 0.001; for *C. orientalis, C. foliascens* and *C. coralliophila*, respectively) and overall, concentrations of Chl a were stable in the 3 phototrophic species throughout the experiment (Fig. 4b). Lower concentrations of Chl a were only detected in *C. orientalis* smothered by sediment for 30 d and *C. coralliophila* for 16 d, although this was not significantly different to the respective controls (Fig. 4b, Table 2B). Non-metric Multi-Dimensional Scaling (nMDS) analysis of normalized data for all pigments retrieved by spectrophotometry (Chl a, Chl b, Chl c, Chl d, total chlorophylls and carotenoids) showed no grouping according to smothering time (Fig. 4c). PERMANOVA analysis detected marginally significant differences between smothering times in *C. foliascens* (Table 2C).

### Microbial community analysis

**Figure 5:**
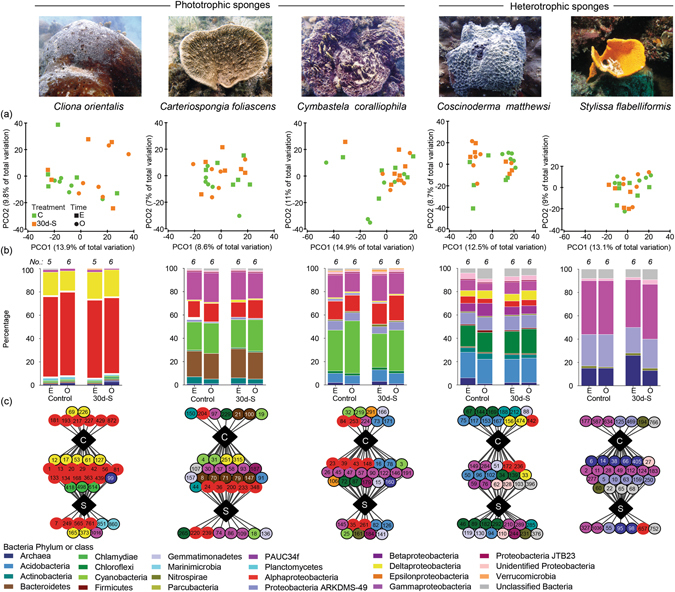
Microbial responses to smothering. (**a**) Principal coordinate analysis plots for control and samples smothered for 30 d (30d-S) at the end of the experimental period (**E**) and 14 d observational period (**O**), (**b**) Average relative abundance of each bacterial phylum (and class for *Proteobacteria*) using OTUs representing greater than 1% of the community for 30d-S vs. control samples across both sampling times, (**c**) Cytoscape networks of the microbial community in all species for controls (**c**) versus 30d-S samples (**S**) across both sampling times.

A total of 9,136,254 high quality 16S rRNA gene amplicon sequences were recovered from the 5 sponge species (n = 169 individual samples) and 6 seawater samples. Each sponge species maintained a unique microbial community (Table 3A, Fig. 5, see also Supplementary Fig. S2) that was distinct from the microbial community of the seawater (Table 3B, Supplementary Figs S2, S3). Aquarium acclimation (for 4 weeks) did not affect the sponge-associated microbial community of any species (Table 3A), although significant differences were observed between field/time 0 and experimental/observational samples in all species (Table 3A). The 30 d smothering treatment did not have a major impact on the overall composition of the sponge microbiome at the phyla level (Fig. 5b), and no significant differences were detected at the OTU level (97% sequence similarity) for any species (Table 3C). In addition, no clear groupings were observed in the ordination (Fig. 5a). For all species, network analyses of the 30 most discriminatory operational taxonomic units (OTUs) for the control sponges and sponges smothered for 30 d, revealed phylogenetically diverse and stable core microbiomes (i.e. the component shared between all samples), as well as treatment- specific OTUs (Fig. 4c, Supplementary Table S2). In each species, the number and taxonomy of OTUs observed exclusively in control samples was generally balanced by the number and taxonomy of new OTUs that were observed only in sediment-exposed samples. Also of note was the finding that core *Cyanobacteria* OTUs in the two photosynthetic species *C. foliascens* and *C. coralliophila* were not disrupted by the sediment exposure.

**Table 3:** PERMANOVA analyses of the microbiome community.

Source	*df*	MS	Pseudo-*F*	*P* (perm)
**(A)**				
Species	4	73933	36.478	0.0001
Time	3	4040.6	1.9936	0.0001
Species × Time	12	2978.2	1.4694	0.0001
Residuals	145	2026.8		
Pair-wise Tests CLI:F ≠ E,O; CAR:F ≠ E ≠ O; CYM: T0 ≠ O; COS: F,T0 ≠ E,O; STY: F ≠ E,O				
**(B)**				
Source	1	23050	5.3963	0.0001
Residuals	169	4271.5		
**(C)**				
***C. orientalis***				
Treatment	1	3316.9	1.23335	0.1431
Time (treatm.)	2	2872.8	1.0684	0.2923
Residuals	18	2689		
***C. foliascens***				
Treatment	1	1989.6	1.0912	0.2612
Time (treatm.)	2	2020.2	1.108	0.1326
Residuals	20	1823.3		
***C. coralliophila***				
Treatment	1	2048.2	1.0691	0.3399
Time (treatm.)	2	1619.3	0.84523	0.854
Residuals	20	1915.8		
***C. matthewsi***				
Treatment	1	2558.5	1.18888	0.1192
Time (treatm.)	2	2774.7	1.2892	0.0205
Residuals	20	2152.2		
***S. flabelliformis***				
Treatment	1	1485.4	0.95496	0.5617
Time (treatm.)	2	1889.1	1.2145	0.0728
Residuals	22	1555.4		

PERMANOVA with (A) species and time as factors, (B) source as factor (sponge host vs. seawater) and, (C) sediment treatment and time (nested to treatment) as fixed factors for all 5 sponge species. In pair-wise tests, F: field control, T0: time 0 control, E: sampling at the end of the exposure time (i.e. 30 d), O: sampling after the 14 d observational period; CAR for *C. foliascens*, CYM for *C. coralliophila*, CLI for *C. orientalis*, COS for *C. matthewsi* and STY for *S. flabelliformis*; Control (C) and smothered (S) within treatments.

## Discussion

All sponges survived the repeated deposition events and subsequent sediment covering, with no partial mortality and few observable physiological effects. To separate cause-effect pathways for dredging related pressures, this experiment focussed on the effects of sediment deposition (i.e. smothering). The SSCs in the experimental tanks were very low once sediments had settled out of suspension, and were typically <1–2 mg L^−1^ for 95% of the exposure periods. All species showed the ability to clear some of the sediment from their surfaces although the self-cleaning capacity varied considerably between species. The highest self-clearance was observed in *C. orientalis* followed by *S. flabelliformis* and *C. foliascens*. Both *C. coralliophila* and *C. matthewsi* were less effective and remained with >85% of their surface covered with sediment for the most of the study.

Sponge morphology provided a passive mechanism for sediment rejection. Erect growth forms are expected to be less affected by sedimentation^20, 22^, and in this experiment, the erect sponge *S. flabelliformis* had some of the highest sediment removal rates. Massive and cup shaped sponge species are prone to sedimentation^19, 20, 22, 43, 44^ consistent with the high sediment covering (>70–80% of their surface) observed here in the massive species *C. matthewsi* and cup morphologies *C. foliascens* and *C. coralliophila*. Other strategies used by sponges to cope with heavy sedimentation rates include sediment sloughing via mucus production (reviewed in ref. 20) and the production of a mucus-like substance was particularly prominent in *C. foliascens* and *S. flabelliformis* (see Supplementary Fig. S1). For vertically oriented planes of tissue, the production and subsequent sloughing of the sediment-impregnated substances resulted in clean underlying tissue surfaces (see Supplementary Fig. S1). The term mucus is used here in a broad sense (see ref. 45) as no attempt was made to identify the nature of the mucus-like layers. The production of similar mucus-like layers (also referred to as sheets, envelopes, webs, films, mats and tunics) which collect sediment and then peel from the surfaces have been described in scleractinian corals (principally *Porites* spp., ref. 46), and appear to play a similar self-cleaning role^10, 47^.

The phototropic, bioeroding sponge *C. orientalis* manipulated and cleaned its surfaces of sediments differently to the other experimental species. *C. orientalis* showed the highest clearance rate, was capable of removing 60−80% of the sediment covering its surface within a few days, and did not lose this ability despite multiple deposition events. *C. orientalis* appears to be able to manipulate settled sediment as evidenced by the discrete sediment patches with well-defined edges, located on otherwise sediment-free tissue. The sediment patches were rarely seen on the sponge periphery and were often located in central hollows or concave areas. These observations suggest that some species are capable of moving and shedding sediment from the edges, as long as the sediment doesn’t become trapped in local depressions (see below). Similar sediment-shedding has been seen in encrusting scleractinian corals associated with muco-ciliary transport^48^, although the mechanism of sediment manipulation in *C. orientalis* is still unclear. Clionaid species have a microhispid surface where spicules protrude from the surface for 0.1–2 mm, and it has been suggested that this creates surface roughness that prevents adherence of dirt and induces passive self-cleaning, repelling debris^20, 40^. For clay and fine silt sized particles (<30 µm) as used in these experiments (and typically encountered in dredging plumes^6^), this bristly surface is more likely to trap and prevent the movement of sediments, rather than repelling them. In scleractinian corals, pulsed tissue contraction is an additional mechanism to mobilize settled sediments and a parallel mechanism may exist for *C. orientalis*.

Sponge respiration rates have been reported to fluctuate greatly in response to elevated suspended sediments^7, 49–51^, but sediment smothering for 30 d did not impact sponge respiration. The lack of significant effects on respiration is consistent with the absence of sponge necrosis or mortality, as opposed to the high mortality rates and bleaching observed in response to high SSC in previous experiments^7^. However, the slight increase in respiration rates observed after 4 d of sediment smothering in most species was concomitant with higher clearance rates and increased mucus production indicating an initial short-term response as sponges activate their cleaning mechanisms to remove sediments. Respiration rates were nevertheless consistent after 16 and 30 d exposure, suggesting a longer term acclimation to sedimentation. Increased respiration rates after short-term acute sediment exposure have been reported in the massive sponge species *Rhopaloeides odorabile* ^51^ and *Coscinoderma matthewsi* ^7^, although decreased respiration rates have been described in other species exposed to chronic levels of high suspended sediments^7, 49, 50^, likely in an attempt to reduce clogging of their aquiferous systems.

Previous research has suggested that whilst sponges with high clearance rates may perform better in environments where there is high sedimentation, this cleaning strategy is likely to be energetically taxing, with longer term implications for energy reserves^20^. In this study there was no clear effect of sediment smothering on total lipid content in any species. However, significantly higher lipid content in smothered versus control *C. orientalis* samples was observed, which may relate to an increase in *Symbiodinium* populations in an attempt to elevate phototrophy, as has been observed in response to high SSC^7^. These results clearly demonstrate that repetitive smothering events for up to 30 d do not cause a decrease in nutritional reserves. This contrasts with previous research assessing the impact of elevated SSCs, where an overall decrease in lipid content, especially among the heterotrophic species was observed^7^. Negative growth rates were observed in most sponges exposed to 16 and 30 d of smothering; however, 30 d of sediment smothering did not cause any total or partial mortality, visible signs of host stress (e.g. bleaching or necrosis), lipid depletion, changes in sponge respiration rates or effects on the microbial composition of any species.

The primary exception to the lack of visible signs of stress was partial discolouration (bleaching) in the phototrophic species *C. orientalis*. As discussed earlier, *C. orientalis* appears capable of clearing sediments from its surface although small patches of sediments were observed in local surface depressions and remained there for extended periods. Significantly higher photosynthetic yields in *C. orientalis* after the 4 d treatment suggested some initial photoacclimation, as reported for other sponges under low light or sediment exposure^8, 19, 52^. However, in *C. orientalis* this photoacclimation could not be maintained in the long term. Lower photosynthetic yields and Chl a concentrations and bleaching were seen in the 16 d and 30 d treatments, and in areas where sediments had overlain the tissues for at least 8 consecutive days. The light attenuating properties of sediment are well known, and light transmission through a 66 mg (dry weight) layer of silt-sized sediments (similar to the exposure conditions used here) is reduced to 0.2–7.7%^42^. Partial sponge bleaching was likely a response to light deprivation under the sediment layer, but once removed there was full recovery of colour in the observational phase. In fact, all phototrophic species experienced total recovery of Chl a in less than 2 d once sediments were removed, as evidenced by hyperspectral and time-lapse imaging. The recovery of bleached areas of *C. orientalis* within ~6 d was facilitated by symbionts rapidly repopulating from unbleached areas, consistent with symbiont translocation patterns previously described in this species^53^.

In addition to changes in photosymbionts, the composition of the sponge microbiome at both the phyla and OTU levels was unaffected by 30 d of sediment smothering, with cyanobacterial OTUs maintained in all sediment-smothered sponges. The only exception to the microbiome stability was a small increase in Archaea in smothered samples of *S. flabelliformis*, and also in water samples from tanks where the sediment-exposures occurred. This result contrasts with previous experiments where the microbial community rapidly shifted in response to other dredging related pressures (i.e. light attenuation). Microbiome shifts are known to destabilise the sponge holobiont and cause subsequent mortality of some species (e.g. *C. foliascens*)^7, 8^. Hence, although sediment smothering does not appear to impact symbiont health and composition, continuous and complete smothering which results in prolonged light attenuation may affect holobiont health in the longer term.

Different sponges have different strategies to cope with sediments and in some cases these can be beneficial, with some species incorporating sediments into their tissues to reinforce outer layers and provide shade^36^, and others living partially embedded in sediments (psammobiosis) to reduce the risk of spongivory^36^. However, sediments can also be detrimental, and previously we have shown that high SSCs alone (in the absence of light attenuation or sediment smothering) resulted in negative sponge growth and decreased respiration rates in most species, and necrosis and mortality within the cup sponge *C. foliascens*, and to a lesser extent in the massive sponge *C. matthewsi* ^7^. In another study, light attenuation (in the absence of elevated SSCs or sediment smothering) impacted the phototrophic species *C. orientalis* and *C. foliascens* ^8^, resulting in tissue bleaching. Although *C. orientalis* exhibited full recovery under normal light conditions, *C. foliascens* did not, showing high levels of mortality^8^. Results from this study, where sediment deposition was isolated as the primary variable, showed that repeated deposition events and near continual covering of the sponges in a sediment layer for an extended period did not cause sponge mortality. Some species seem capable of existing partially or fully covered in sediment i.e. *C. coralliophila* and *C. matthewsi*, while other species possess an ability to self-clean, i.e. they have active sediment-rejection mechanisms. However, when these mechanisms become overwhelmed and sediments build-up, localized reductions in Chl a concentrations or partial bleaching can occur, e.g. *C. orientalis*. The cause-effect pathway is likely to involve light limitation, and the effect is rapidly reversible upon removal of the sediment. In conjunction with previous research on the impacts of light attenuation^8^ and SSC^7^, thresholds can now be used in water quality monitoring programs to alert dredging proponents to levels of light reduction, SSC and sedimentation that, if continued, could detrimentally impact sponge populations.

Relating these findings to conditions that can occur *in situ* during dredging programs, or natural wind and wave induced sedimentation events is difficult because of the problems associated with accurately measuring sediment deposition rates in shallow benthic environments at the appropriate sensitivity and temporal scale (i.e. mg cm^−2^ over hours or days, reviewed in ref. 54). Sediment traps have been used extensively to estimate ‘sedimentation’ rates on reefs but are more apt to record information on suspended-sediment dynamics than to provide any useful data on sedimentation *per se* ^55^. Two recent studies have measured sediment deposition rates at a highly turbid site in the inshore zone of the Great Barrier Reef. The techniques used which involved shallow trays^56^ and a newly configured sediment deposition sensor^57^, do not suffer from the resuspension-limitation and deposition-bias associated with traps. Estimates of net sedimentation were 3–7 mg cm^−2^ d^−1^ (over the course of a year)^56^ and ~8 mg cm^−2^ d^−1^ (during typical conditions where SSCs ranged from 1−28 mg L^−1^)^57^. The deposition rates in this experiment (30−45 mg cm^−2^ d^−1^) were up to 6× higher than levels found in the highly turbid inshore reef system. Experimental deposition events initially resulted in 80–100% of the sponges surface being covered with a 0.5 mm thick layer of sediment and sponges in some treatments were exposed to up to 7 such deposition events over an extended 30 d period. We consider the exposure regimes in terms of intensity, duration and frequency to be extreme compared to what can occur naturally *in situ* and the resulting smothering of the sponges is consistent with *in situ* observations of smothering of benthic organisms close to dredging projects.

In summary, exposure over an extended period to multiple discrete sediment deposition events resulting in a near continuous covering of sediment on their surface did not alter the health of most adult sponges. Sponges possessed either active or passive self-cleaning strategies which allowed them to continue feeding, and were able to tolerate quite high levels of partial covering of their surfaces by sediment. These experiments were designed to examine a specific cause-effect pathway associated with sediment deposition alone. In reality, during dredging, sponges would be exposed to sediment deposition in combination with elevated SSCs and the associated light reduction from high turbidity. The results from this study will support the interpretation of experiments examining the combined effects of all three dredging-related pressures and aid the subsequent development of water quality thresholds for impact prediction purposes. Further research is also required to assess the effects of those stressors on reproduction and the early life-history stages, as higher vulnerability and reduced energy reserves available for those processes would have long-term consequences for sponge population dynamics.

## Methods

### Sample collection

This study used the phototrophic species *Cliona orientalis* Thiele, 1900, *Carteriospongia foliascens* (Pallas, 1766) and *Cymbastela coralliophila* Hooper & Berquist 1992 and the heterotrophic species *Coscinoderma matthewsi* (Lendenfeld, 1886) and *Stylissa flabelliformis* (Hentschel, 1912)^22, 30, 58–60^ to examine responses across different nutritional modes. The selected species are common throughout the Indo-Pacific, including the east and west coasts of tropical Australia, represent different morphologies and nutritional modes (Supplementary Table S3) and perform well in aquaria under experimental conditions^22^. All sponges were collected from 3–15 m depth from the Palm Islands, central Great Barrier Reef (GBR) (Supplementary Table S3). *C. orientalis* is an encrusting species that bioerodes coral, so cores of *C. orientalis* were air-drilled from dead colonies of *Porites* sp. ensuring > 2 cm of coral substrate below the sponge. For all species, sponges were cut into similar sized explants (~5 × 5 cm), and acclimated under natural light in flow-through seawater for 4 weeks until fully healed.

### Experimental set up

The experiment was performed in the National Sea Simulator (SeaSim) at the Australian Institute of Marine Science (AIMS, Townsville, Queensland, Australia) using 150 L clear PVC acrylic tanks supplied with a continuous inflow of 5 µm filtered seawater at a rate of 600 mL min^−1^ to ensure sponges received sufficient particulate and dissolved food. The seawater in experimental tanks was set to 27 °C, representing the temperature at the time of sponge collection. Experimental treatments comprised smothered v control tanks (in which no sediments were added) and 3 different exposure times (4, 16 and 30 d, see Fig. 1a). In the smothered treatments, sponges received repeated doses of sediment slurry every ~4 d, sufficient to cause continuous smothering (~30−45 mg cm^−2^). A VorTech™ MP10 propeller pump (EcoTech Marine, PA, US) was left on for 10 min after sediment was added to ensure homogenous turbidity throughout the tank. Pumps were then turned off for 24 h, to allow all sediments to settle out of suspension onto the sponges. After the sediments had settled, the pump was re-started to provide sufficient water flow to the sponges without lifting the sediments from the sponges’ surfaces. The calcareous sediment used in this experiment was collected from the lagoon of Davies Reef, a mid-shelf reef centrally located in the GBR (S 18° 49.354′ E 147°38.253′). Sediments were first screened to 2 mm and then ground with a rod mill grinder until the mean particle size was 29 µm (with 80% of the sediment 3–64 µm, typical for dredge plumes^6^), measured using laser diffraction techniques (Mastersizer 2000, Malvern instruments Ltd, UK).

Tanks were illuminated by AI Hydra FiftyTwo™ HD LED lights (Aquaria Illumination, IA, US) on a 13:11 h L:D cycle, with a light regime designed to simulate daily conditions on the reef^3, 61^. Each day there was a 6 h ramp up to a maximum instantaneous light level of 200 µmol photons m^−2^ s^−1^, followed by 1 h of constant illumination, and a 6 h ramp down to full darkness, resulting in a daily light integral (DLI) of 5 mols photons m^−2^. Following the treatment period (4, 16 or 30 d), the surface of all sponges was gently brushed to remove any deposited sediments and all sponges were further exposed to clear seawater for a 14 d observation period (i.e. recovery, see Fig. 1a). Each treatment comprised 3 replicate tanks containing 4 sponge replicates per species (i.e. n = 12 replicates of each species per treatment).

### Studied parameters

The effect of sediment smothering on the sponge holobiont was determined using a suite of response variables, with a particular focus on changes in sponge feeding activity and the associated symbiotic microbiome. To obtain baseline data on sponge health, 6 extra individuals were processed for each species immediately after collection (field controls) and after aquarium acclimation (t = 0 controls). Unless otherwise stated, statistical analyses and graphs were performed using the software SigmaPlot v.11.0 (Systat Software Inc.) and R^62^. Linear mixed models using tank as a random factor and treatment and time (B, E and O) as fixed factors were fitted by residual maximum likelihood (REML) for data pertaining to relative growth rates, respiration, lipid content, chlorophyll fluorescence and total pigments. For sediment clearance rates, the same method was used but with time as the only fixed factor. For analyses with repeated measures of the same individual, i.e. clearance rates, growth, respiration rates, and chlorophyll fluorescence, individual was also included as a random factor. An analysis of variance (ANOVA) table was generated for each model and Tukey’s post–hoc multiple comparisons were performed to compare treatment levels for each model^63^.

### Physical parameters

In order to measure sedimentation rates (SR), two SedPods^64^ (surface area = 25.16 cm^2^) were placed in each tank before sediment addition and removed after the 24 h sedimentation period. SR were then measured by collecting and filtering sediments that accumulated on the SedPods using gravimetric techniques (filtration through 0.4 µm polycarbonate filters and calculating dry weight of the filters). Turbidity was monitored throughout the experiment using a manual WP 88 Turbidity Meter (TPS). Differences in sedimentation between replicate tanks and day of sediment pulse were assessed using two-way analysis of variance (ANOVA) with tank and time as fixed factors. A one-way ANOVA was used to study differences in the turbidity levels between replicate tanks (fixed factor) throughout the experiment.

### Sediment smothering and clearance rates

Sponge surface area covered by sediments and sediment removal (i.e. clearance rates) were recorded throughout the experiment using a digital camera with underwater housing (Canon S120) and analysed using image analysis software (ImageJ^65^).

### Growth and necrosis

Initial and final thickness (in *C. foliascens*, *C. coralliophila* and *S. flabelliformis*), height (in *C. matthewsi*), or sponge tissue depth inside coral cores (in *C. orientalis*), of each sponge (±0.1 mm) was measured as a proxy for growth. Callipers were used to take three measurements per sponge and averaged to calculate percentage change throughout the experiment and during the subsequent observational period. Partial mortality (necrosis) and loss of photosynthetic symbionts (bleaching) was recorded pre and post sediment exposure and at the end of the observational phase, using a digital camera (Canon S120) with underwater housing and analysed using ImageJ^65^.

### Respiration rates

Changes in sponge respiration rates were measured throughout the experiment and observational period. Samples were dark adapted for a minimum of 30 min before being transferred to 600 mL respiration chambers where they were incubated for 30 min at 27 °C. Constant mixing within the chambers was achieved using a magnetic stir bar and a submergible battery operated platform. An HDQ30D flexi meter (HACH LDO™, CA, US) was used to take 3 initial readings (i.e. initial O_2_) and to measure the mg L^−1^ of O_2_ and the % O_2_ in each chamber at the end of the incubation. The final oxygen concentration inside the chamber did not drop below 85% saturation in most cases. To control for microbial community respiration, a chamber containing only seawater (blank) was incubated under identical conditions. In order to normalize oxygen consumption among individuals, sponge volume was estimated by multiplying the surface area of each individual (derived from photos taken simultaneously and analysed using ImageJ) by its average thickness or height (as explained above). The same sponge volume was used to account for differences in water volume within the respiration chambers. In the case of *C. orientalis*, the height of the sponge layer within the coral cores was used to normalize oxygen consumption per sponge volume, whereas the height of the whole core (including the coral substrate) was used to calculate the specific volume of water within the chambers. Respiration data from *C. coralliophila* and *S. flabelliformis* were log and cube root transformed, respectively, in order to meet assumptions of homogeneity of variance.

### Lipid analysis

The concentration of total lipids in sponge tissue was measured over time to assess whether sediment smothering interferes with sponge feeding. Samples were analysed from controls at the start of the experiment (T = 0), the 30 d treatment and its respective control, at the end of the exposure time and at the end of the observational period. Approximately 3 cm^3^ of sponge tissue was excised, wrapped in aluminium foil (to prevent plasticizer contamination) and immediately frozen in liquid nitrogen. Lipids were extracted from approximately 100 mg of sample as described in ref. 66 and following modifications in ref. 67, with total lipid content reported as percentage biomass based on a dry weight conversion factor.

### Hyperspectral imaging

Hyperspectral imaging can be used to non-destructively estimate relative chlorophyll content *in situ* (e.g. refs 68, 69). A Pika II hyperspectral imaging camera (Resonon, MT, US), mounted on a moveable rig (Supplementary Fig. S4) captured back-reflected light in 480 spectral bands (~1 nm resolution) over the range of ~430–900 nm. Based on the methodologies described in refs 68, 70, the 3 phototrophic species, *C. orientalis, C. foliascens* and *C. coralliophila* were scanned after 8 d of being covered with sediment including i) before the sediment was removed, ii) after the sediment was removed and iii) after one day of recovery (n = 3). Scans were compared to control groups (n = 3) and the sponge’s chlorophyll content was estimated by considering the logarithmic difference of the reflectance at chlorophyll absorption maximum (670 nm) and edge (745 nm). This calculation was performed at each pixel, and the values averaged over the imaged area of the specimen. Wavelength standards were selected based on optimal levels for control sponges, as each species had different chlorophyll content. One-way ANOVAs were performed to analyse changes in relative chlorophyll content for each species, separately. Significant differences were tested using pairwise, multiple Holm-Sidak comparisons. For *C. foliascens*, the ANOVA was performed on rank data to meet the assumption of homogenous variance, and a Tukey test was performed for *post hoc* comparison.

Based on the quick recovery of *C. orientalis* in the hyperspectral imaging trials, recovery was monitored using standard time-lapse photography. A partially bleached individual was monitored for 6 d after being covered with sediment for 30 d, with images taken every min.

### Chlorophyll fluorescence

Changes in photosynthetic capacity (maximum quantum yield) of the sponge’s phototrophic symbionts were measured with a Diving-PAM (pulse amplitude modulation) chlorophyll fluorometer (Heinz Walz GmbH, Effeltrich, Germany) for *C. foliascens*, *C. coralliophila* and *C. orientalis*. Chlorophyll fluorescence measurements were taken 10 mm from the sponge tissue using a rubber space between the sponge and the end of the 6 mm fibre-optic probe. Sponges were dark-adapted for 30 min before measurement, and the initial fluorescence (*F*_o_) determined using a pulse-modulated red measuring light (655 nm, ~0.15 µmol photons m^−2^ s^−1^), and maximum fluorescence (*F*_m_) then measured following a saturating pulse of light. Maximum quantum yield (*F*_v_/*F*_m_) was calculated from the ratio of variable fluorescence (*F*_m_-*F*_o_) to maximum fluorescence^71^. Three measurements were obtained before and after the sediment exposure time and at the end of the observational phase.

### Pigment analysis

Pigment analyses were performed on tissue from all phototrophic sponges (*C. foliascens*, *C. coralliophila* and *C. orientalis*) at the end of the experimental time (4, 16 and 30 d) and after the 14 d observational phase. Pigments from samples incorporating pinacoderm and mesohyl regions were extracted and analysed as described in ref. 22 and standardized to sponge wet weight. The concentration of Chlorophyll a (hereafter Chl a) was used as a proxy for changes in photosymbiont health/bleaching^30^. Pearson correlations were performed between all the studied pigments. All pigments retrieved by spectrophotometry (i.e. Chl a, b, c, d, total chlorophylls and carotenoids) were used to build resemblance matrices based on normalized data for each species, separately. Non-metric Multi-Dimensional Scaling (nMDS) plots were created using Euclidean distances. Two factors were determined (i.e. treatment and sampling time, nested to treatment) and examined by PERMANOVA (Permutational multivariate ANOVA based on distances). All multivariate analyses were performed using PRIMER 6 (Primer-E Ltd, UK).

### Microbial community analysis

Microbial community composition was assessed using Illumina amplicon sequencing of the 16S rRNA taxonomic marker gene. Microbial analysis was performed on field controls, sponges collected at the start of the experiment, and at the end of the experimental and observational period for sponges smothered by sediments for 30 d and their corresponding controls. All samples were immediately frozen in liquid nitrogen and subsequently stored at −80 °C. Water samples were simultaneously collected from each tank to facilitate a direct comparison with microbes present in the surrounding environment. DNA was extracted from ~0.2 g of sponge tissue using the PowerPlant® Pro DNA Isolation Kit (MoBio Laboratories, CA, US) according to the manufacturer’s protocol. Microbial communities in seawater were filtered and their DNA extracted as previously described^72^. Sequencing of the 16S rRNA gene was performed at the Australian Centre for Ecogenomics using primers 515 f and 806r and the HiSeq2500 platform with the V2 chemistry (2 × 250 bp) (Illumina). Sequence data was deposited at the NCBI under the accession number SRP080232.

Amplicon sequence data was processed in Mothur v.1.35.1^73^ according to the MiSeq standard operating procedure^74^. Briefly, demultiplexed fastq paired-end reads were first quality-filtered and assembled into contigs (make.contigs and screen.seqs: maxambig = 0, maxhomop = 8, minlength = 100, maxlenght = 292). Aligned reads were reduced to non-redundant sequences and chimeric sequences were detected using Uchime^75^. Aligned sequences were phylogenetically classified based on the Silva reference file v.123, and all undersigned sequences removed (taxon = Chloroplast-Mitochondria-Unkown-Eukaryota). Samples with low read numbers were eliminated from the dataset. The remaining samples were sub sampled to 8,691 sequences. Pairwise distances were calculated and used for clustering and OTU assignment and OTUs were further classified based on the SILVA v.123 taxonomy.

OTU data was normalised to account for sampling depth and then square-root transformed to reduce the effect of abundant OTUs. Bray-Curtis distance matrices were constructed and visualised using non-metric multidimensional plots (nMDS) and principal coordinates analyses (PCO). Permutational analysis of variance (PERMANOVA, using 9,999 permutations) was used to determine significant differences in microbial communities based on source (sponge versus environmental control), time of sampling (field controls, time 0 controls, experimental and observational period samples), and treatment (control versus 30 d smothered). All multidimensional statistical analyses were performed in PRIMER 6/PERMANOVA. Similarity Percentage Analysis (SIMPER) was used to determine the OTUs that contribute to the differences between 30 d smothered and control samples for each species, separately. The 30 OTUs with the most discriminating power from the SIMPER analysis were used to create networks in Cytoscape 3.2.0 (www.cytoscape.org)^76^.

## Electronic supplementary material

Supplementary Information

Supplementary Dataset 1
