# Supplementary material for: Effects of sediment smothering on the sponge holobiont with implications for dredging management
Source: Sci Rep. 2017 Jul 14;7:5156. doi: 10.1038/s41598-017-05243-x (PMC5511226; doi:10.1038/s41598-017-05243-x)

Supporting Online Material

Effects of sediment smothering on the sponge holobiont with implications for dredging management

Mari-Carmen Pineda[1,2], Brian Strehlow[1,2,3], Miriam Sternel[4], Alan Duckworth[1,2], Joost den Haan[5], Ross Jones[1,2] and Nicole S. Webster[1,2]

[1] Australian Institute of Marine Science (AIMS), Townsville, QLD and Perth, WA, Australia

[2]Western Australian Marine Science Institution, Perth, WA, Australia

[3]Centre for Microscopy Characterisation and Analysis, School of Plant Biology, and Oceans Institute, University of Western Australia, Crawley, WA, Australia

[4]University of Bremen, Bremen, Germany

[5]Max Plank Institute for Marine Microbiology, Bremen, Germany

*Corresponding author:

Mari-Carmen Pineda

Australian Institute of Marine Science, PMB3, Townsville, QLD, 4810, Australia

E-mail: mcarmen.pineda@gmail.com.

Tel.: +61 7 4753 4522, fax: +61 7 4772 5852

Table S1. One-way ANOVA comparing relative chlorophyll content over time in smothered sponges immediately post cleaning, after 24h recovery and in control sponges. The ANOVA for *C. foliascens* was performed on ranks. Pairwise Multiple Comparison Procedures: Tukey Test for *C. foliascens*, and Holm-Sidak method for *C. orientalis* and *C. coralliophila*.

		Cliona orientalis		*Carteriospongia foliascens*		*Cymbastela coralliophila*	
Source	df	*F*	*P*	*H*	*P*	*F*	*P*
Treatment<br>Error	3<br>8	38.693	<0.001	9.974	0.019	335.839	<0.001
Pairwise Multiple Comparisons:		Control > Sed. removal (*P*=0.016)		Control > Sed. removal (*P*<0.05)		Control > Sed. removal (*P*=0.002)	

Table S2. Similarity Percentage Analysis (SIMPER) for 30 most significant OTUs driving differences between controls and 30 d smothered samples combining experimental and observational periods.

OTU	Average relative abundance (%)		Contribution (%)	Taxonomic ID
	Control	30d-S		
Cliona orientalis				
Otu000001	6.05	3.86	2.59	Alphaproteobacteria
Otu000007	0.49	2.26	2	Alphaproteobacteria
Otu000020	0.96	1.76	1.6	Alphaproteobacteria
Otu000012	0.86	1.77	1.57	Deltaproteobacteria
Otu000017	1.27	1.8	1.44	Deltaproteobacteria
Otu000029	1.13	1.12	1.14	Alphaproteobacteria
Otu000013	1.67	1.69	1.04	Alphaproteobacteria
Otu000042	1.2	0.51	0.93	Alphaproteobacteria
Otu000061	1.04	0.65	0.91	Deltaproteobacteria
Otu000056	0.57	1.08	0.9	Alphaproteobacteria
Otu000053	0.64	1.25	0.88	Deltaproteobacteria
Otu000069	0.81	0.36	0.65	Deltaproteobacteria
Otu000081	0.78	0.49	0.62	Alphaproteobacteria
Otu000226	0.73	0.06	0.59	Deltaproteobacteria
Otu000131	0.32	0.56	0.59	Alphaproteobacteria
Otu000134	0.58	0.71	0.54	Alphaproteobacteria
Otu000165	0.09	0.69	0.53	Deltaproteobacteria
Otu000133	0.74	0.34	0.53	Alphaproteobacteria
Otu000174	0.33	0.5	0.52	Deltaproteobacteria
Otu000127	0.69	0.5	0.51	Deltaproteobacteria
Otu000192	0.53	0.34	0.51	Alphaproteobacteria
Otu000138	0.53	0.3	0.5	Deltaproteobacteria
Otu000152	0.41	0.37	0.5	Deltaproteobacteria
Otu000181	0.49	0.38	0.45	Alphaproteobacteria
Otu000274	0.17	0.5	0.45	Deltaproteobacteria
Otu000193	0.56	0.15	0.42	Alphaproteobacteria
Otu000217	0.58	0.21	0.42	Alphaproteobacteria
Otu000168	0.29	0.63	0.39	Alphaproteobacteria
Otu000304	0.19	0.32	0.38	Bacteroidetes
Otu000227	0.56	0.37	0.38	Alphaproteobacteria
Carteriospongia foliascens				
Otu000004	2.58	3.02	1.56	Cyanobacteria
Otu000019	1.39	0.86	1.14	Cyanobacteria
Otu000018	1.11	1.53	1.1	Cyanobacteria
Otu000021	1.28	0.77	0.86	Bacteroidetes
Otu000037	1.6	0.61	0.86	Gammaproteobacteria
Otu000008	2.51	2.3	0.84	Bacteroidetes
Otu000031	1.21	1.29	0.73	Cyanobacteria
Otu000030	1.08	1.16	0.71	Gammaproteobacteria
Otu000024	1.51	1.27	0.67	Alphaproteobacteria

Otu000052	0.58	0.86	0.62	Bacteroidetes
Otu000074	0.76	0.96	0.61	Gammaproteobacteria
Otu000044	1.06	1.25	0.58	Actinobacteria
Otu000086	0.5	1.17	0.57	Gammaproteobacteria
Otu000113	0.72	0.49	0.57	Alphaproteobacteria
Otu000054	0.8	0.55	0.55	Gammaproteobacteria
Otu000111	0.64	0.77	0.53	Alphaproteobacteria
Otu000036	1.57	1.3	0.52	Alphaproteobacteria
Otu000058	0.85	1.26	0.51	Gammaproteobacteria
Otu000100	0.94	0.56	0.51	Bacteroidetes
Otu000109	0.76	0.73	0.5	Gammaproteobacteria
Otu000097	0.71	0.73	0.5	Gammaproteobacteria
Otu000070	0.84	1.06	0.5	Bacteroidetes
Otu000093	0.86	0.73	0.47	Gammaproteobacteria
Otu000071	0.87	0.98	0.45	Bacteroidetes
Otu000175	0.4	0.64	0.45	Bacteroidetes
Otu000121	0.37	0.58	0.44	Bacteroidetes
Otu000116	0.73	0.39	0.4	Cyanobacteria
Otu000136	0.46	0.68	0.4	Gemmatimonadetes
Otu000214	0.52	0.35	0.39	Gammaproteobacteria
Otu000158	0.27	0.54	0.39	Alphaproteobacteria
Cymbastela coralliophila				
Otu000003	3.91	3.05	2.32	Cyanobacteria
Otu000009	0.95	1.15	1.3	Cyanobacteria
Otu000032	1.16	0.87	1.19	Cyanobacteria
Otu000035	0.68	1.41	1.09	Alphaproteobacteria
Otu000025	0.65	1.18	1.02	Cyanobacteria
Otu000041	0.82	0.78	0.98	Cyanobacteria
Otu000015	2.04	1.77	0.9	Gemmatimonadetes
Otu000026	1	1.6	0.84	Gammaproteobacteria
Otu000016	1.43	1.73	0.77	Acidobacteria
Otu000023	1.4	1.63	0.76	Alphaproteobacteria
Otu000039	0.81	1.26	0.76	Alphaproteobacteria
Otu000077	0.64	0.63	0.74	Cyanobacteria
Otu000115	0.74	0.23	0.68	Cyanobacteria
Otu000101	0.76	0.52	0.67	Cyanobacteria
Otu000064	0.68	0.44	0.66	Cyanobacteria
Otu000002	0.68	0.25	0.66	Gammaproteobacteria
Otu000045	0.87	1.33	0.63	Gammaproteobacteria
Otu000043	1.06	1.09	0.63	Alphaproteobacteria
Otu000084	0.76	0.67	0.61	Alphaproteobacteria
Otu000073	1.01	0.72	0.59	Acidobacteria
Otu000080	0.55	0.65	0.57	Alphaproteobacteria
Otu000072	0.88	0.89	0.57	Chloroflexi
Otu000118	0.58	0.39	0.53	Gemmatimonadetes
Otu000219	0.67	0.07	0.53	Cyanobacteria
Otu000132	0.48	0.6	0.53	Gammaproteobacteria
Otu000082	0.55	0.8	0.52	Acidobacteria
Otu000126	0.41	0.79	0.52	Acidobacteria
Otu000078	0.75	0.94	0.52	Acidobacteria
Otu000090	0.75	0.91	0.52	Gammaproteobacteria
Otu000057	0.81	0.67	0.48	Gammaproteobacteria
Coscinoderma matthewsi				
Otu000034	1.32	1.15	0.4	Chloroflexi
Otu000046	0.67	1.19	0.38	Chloroflexi
Otu000040	0.73	0.89	0.36	Acidobacteria
Otu000050	0.84	0.97	0.36	Acidobacteria
Otu000088	1.21	0.47	0.35	Gemmatimonadetes
Otu000033	0.97	1.61	0.34	Deltaproteobacteria
Otu000119	0.12	0.89	0.32	Gemmatimonadetes
Otu000089	0.39	0.82	0.3	Chloroflexi

Otu000075	0.85	0.62	0.29	Acidobacteria
Otu000067	0.85	0.58	0.28	Chloroflexi
Otu000128	0.57	0.51	0.28	Unclassified Bacteria
Otu000103	0.91	0.66	0.28	Unclassified Bacteria
Otu000094	0.66	0.79	0.28	Acidobacteria
Otu000144	0.9	0.35	0.27	Chloroflexi
Otu000051	0.99	0.94	0.26	Gemmatimonadetes
Otu000117	0.74	0.32	0.26	Acidobacteria
Otu000076	0.95	0.9	0.24	PAUC34f
Otu000140	0.34	0.52	0.24	Unclassified Bacteria
Otu000108	0.23	0.53	0.24	Chloroflexi
Otu000195	0.69	0.3	0.24	Acidobacteria
Otu000142	0.74	0.55	0.24	Alphaproteobacteria
Otu000156	0.72	0.29	0.23	Deltaproteobacteria
Otu000059	0.84	0.82	0.23	PAUC34f
Otu000153	0.71	0.55	0.23	Acidobacteria
Otu000096	0.7	0.83	0.23	Acidobacteria
Otu000203	0.64	0.37	0.22	Gemmatimonadetes
Otu000212	0.71	0.34	0.22	Actinobacteria
Otu000234	0.56	0.24	0.21	Acidobacteria
Otu000189	0.59	0.4	0.21	Acidobacteria
Otu000110	0.54	0.68	0.21	Unidentified Proteobacteria
Stylissa flabelliformis				
Otu000006	2.12	2.61	2.31	Archaea
Otu000014	1.03	1.23	1.78	Archaea
Otu000022	1.32	1.41	1.48	Unclassified Bacteria
Otu000028	1.52	1.05	1.44	Gammaproteobacteria
Otu000011	1.5	1.97	1.36	Gammaproteobacteria
Otu000002	5.2	4.69	1.3	Gammaproteobacteria
Otu000027	0.86	1.25	1.08	Unidentified Proteobacteria
Otu000049	0.74	0.92	1.08	Gammaproteobacteria
Otu000068	0.77	0.8	1.06	Unclassified Bacteria
Otu000055	0.6	0.95	1.04	Proteobacteria ARKDMS-49
Otu000083	0.51	0.66	1.03	Gammaproteobacteria
Otu000066	0.82	0.84	0.9	Archaea
Otu000095	0.43	0.66	0.88	Archaea
Otu000060	0.7	1.07	0.86	Nitrospirae
Otu000065	0.78	0.75	0.83	Unclassified Bacteria
Otu000085	0.48	0.29	0.76	Unclassified Bacteria
Otu000159	0.63	0.47	0.74	Proteobacteria ARKDMS-49
Otu000098	0.41	0.56	0.74	Archaea
Otu000038	1.05	1.19	0.7	Archaea
Otu000112	0.83	0.71	0.68	Gammaproteobacteria
Otu000176	0.38	0.4	0.66	Gammaproteobacteria
Otu000125	0.5	0.38	0.65	Proteobacteria ARKDMS-49
Otu000005	3.81	3.38	0.64	Proteobacteria ARKDMS-49
Otu000194	0.6	0.37	0.6	Nitrospirae
Otu000129	0.36	0.38	0.55	Gammaproteobacteria
Otu000163	0.45	0.36	0.54	Unclassified Bacteria
Otu000124	0.52	0.7	0.51	Gammaproteobacteria
Otu000010	2.39	2.3	0.5	Proteobacteria ARKDMS-49
Otu000177	0.5	0.26	0.49	Gammaproteobacteria
Otu000242	0.4	0.03	0.48	Gammaproteobacteria

Table S3. Sponge sampling details. List of species, morphologies, nutritional mode and sampling location.

Species Name (Author)	Functional Morphology	Primary Nutritional Mode	Sampling Location
Cliona orientalis Thiele, 1900	Encrusting (bioeroding)	Phototrophic [63]	Pelorus Is. (Palm Is.) S 18°32.903' E 146° 29.172'
Carteriospongia foliascens (Pallas, 1766)	Cup (wide cup)	Phototrophic [62]	Fantome Is. (Palm Is.) S 18°41.028' E 146° 30.706'
Cymbastela coralliophila Hooper & Bergquist, 1992	Encrusting (thick) Cup (table)	Phototrophic [64]	Pelorus Is. (Palm Is.) S 18°32.903' E 146° 29.172'
Coscinoderma matthewsi (Lendenfeld, 1886)	Massive	Heterotrophic (Wilkinson 1983)	Pelorus Is. (Palm Is.) S 18°32.903', E 146° 29.172'
Stylissa flabelliformis (Hentschel, 1912)	Erect (laminar)	Heterotrophic [26]	Pelorus Is. (Palm Is.) S 18°32.903' E 146° 29.172'

Figure S1. Photographs of the 5 sponge species before sediment addition, during sediment smothering and after sediment removal. Phototrophic species: a) *C. orientalis*, b) *C. foliascens*, c) *C. coralliophila*, and Heterotrophic species d) *C. matthewsi* and d) *S. flabelliformis*.

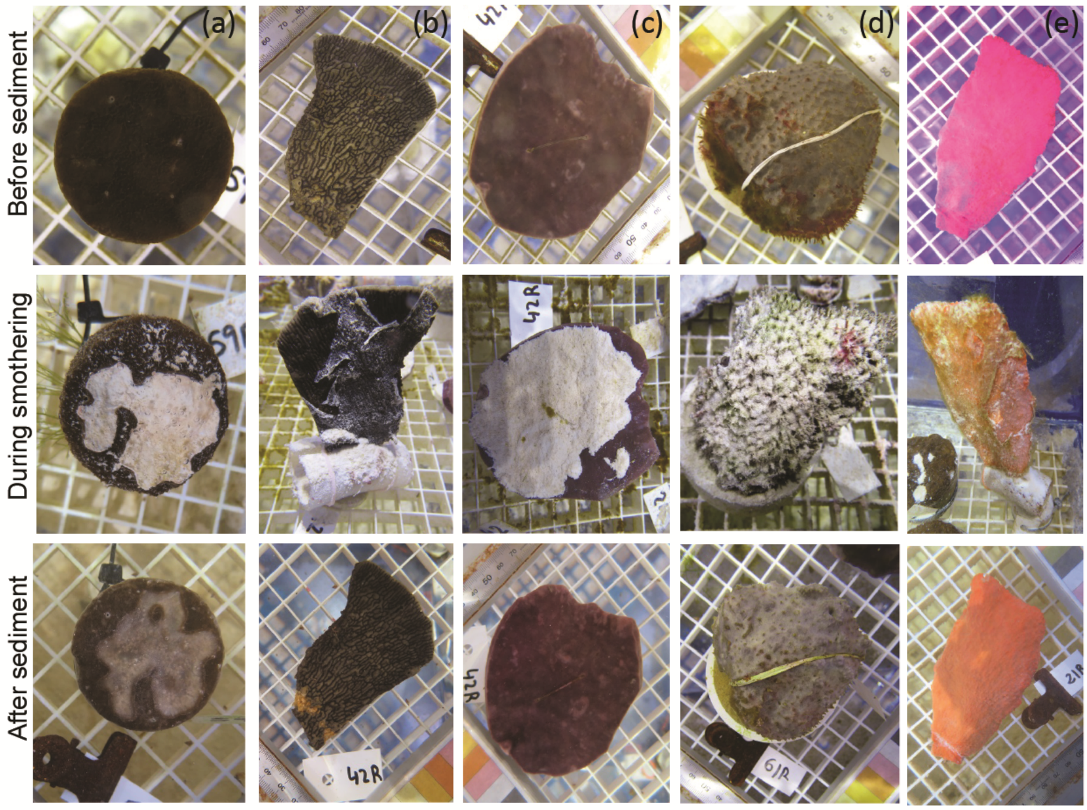

Figure S2. Non-metric Multi-Dimensional Scaling plots on microbial OTU data. nMDS of microbial communities for all 5 sponge species and the environmental control (seawater).

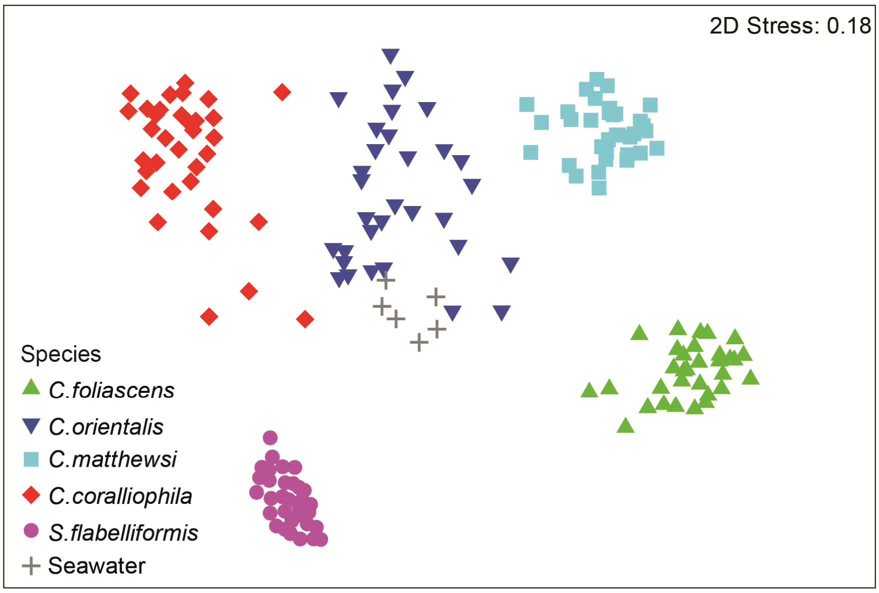

Figure S3. Phylum level bar chart for seawater (environmental control). Average relative abundance of each bacteria phylum (and class for Proteobacteria) for 3 replicate seawater samples within each targeted treatment (Control versus 30-days smothered). Only OTUs representing greater than 1% of the overall community were included.

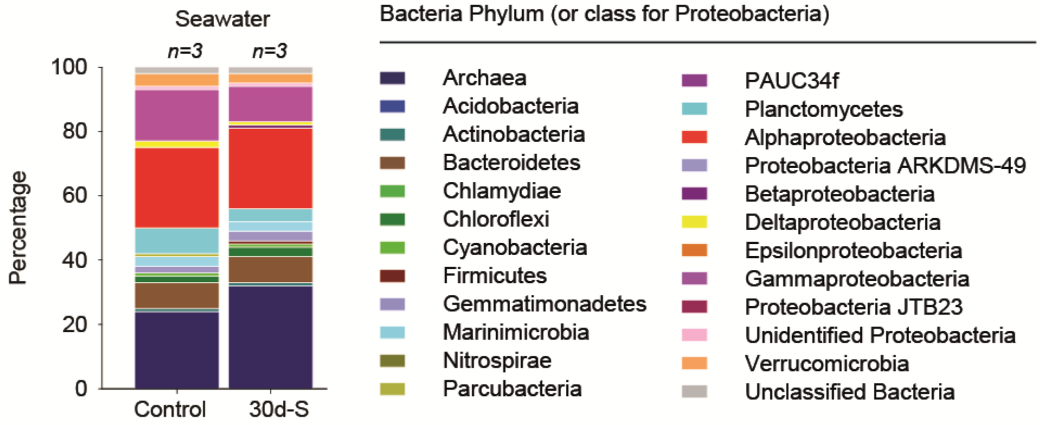

Figure S4. Hyperspectral imaging setup. The Hyperspectral camera (C) moves along a track (i.e. left to right), capturing the back-reflected light off the sponges to quantify chlorophyll concentrations *in situ* (based on the relative chlorophyll content). The external light (i.e. a clean white light spectrum) is provided by two halogen lights (L). Whilst incubated, the 3 sponge species (S) can be scanned simultaneously.

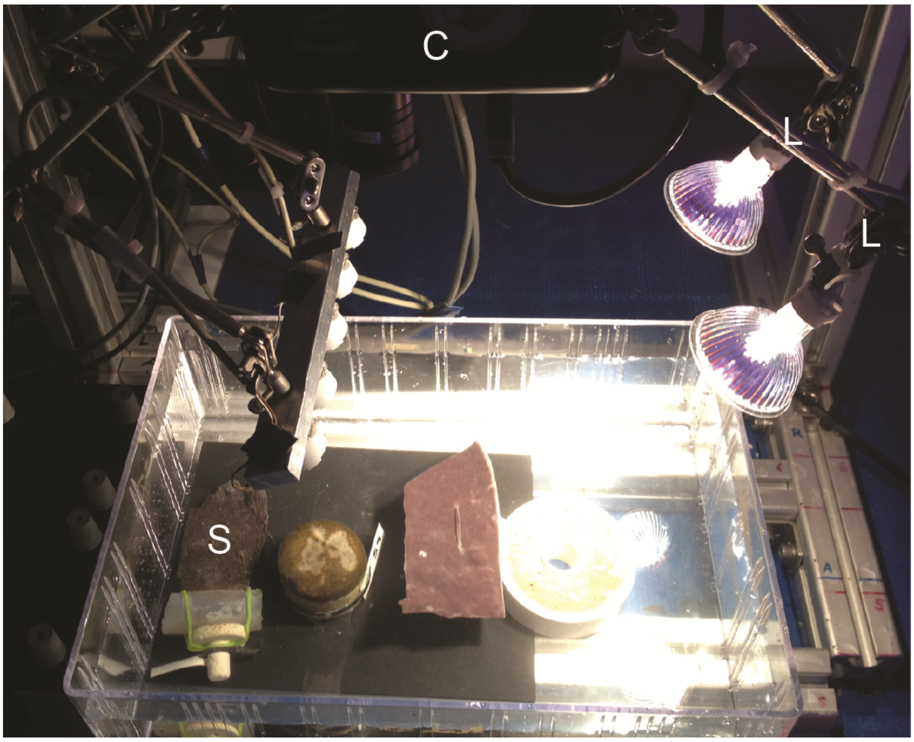

Supplement: Supplementary file 1 — Supplementary Information [file 41598_2017_5243_MOESM1_ESM.pdf]
